# Topological network features determine convergence rate of distributed average algorithms

**DOI:** 10.1038/s41598-022-25974-w

**Published:** 2022-12-17

**Authors:** Christel Sirocchi, Alessandro Bogliolo

**Affiliations:** 1 Dipartimento di Scienze Pure e Applicate, Università degli Studi di Urbino, 61029 Urbino, Italy; 2 DIGIT srl, 61029 Urbino, Italy

**Keywords:** Engineering, Mathematics and computing

## Abstract

Gossip algorithms are message-passing schemes designed to compute averages and other global functions over networks through asynchronous and randomised pairwise interactions. Gossip-based protocols have drawn much attention for achieving robust and fault-tolerant communication while maintaining simplicity and scalability. However, the frequent propagation of redundant information makes them inefficient and resource-intensive. Most previous works have been devoted to deriving performance bounds and developing faster algorithms tailored to specific structures. In contrast, this study focuses on characterising the effect of topological network features on performance so that faster convergence can be engineered by acting on the underlying network rather than the gossip algorithm. The numerical experiments identify the topological limiting factors, the most predictive graph metrics, and the most efficient algorithms for each graph family and for all graphs, providing guidelines for designing and maintaining resource-efficient networks. Regression analyses confirm the explanatory power of structural features and demonstrate the validity of the topological approach in performance estimation. Finally, the high predictive capabilities of local metrics and the possibility of computing them in a distributed manner and at a low computational cost inform the design and implementation of a novel distributed approach for predicting performance from the network topology.

## Introduction

### Distributed averaging

In the past two decades, the exponential increase in connected devices has determined a paradigm shift from centralised to highly distributed systems. Distributed computation has drawn considerable attention for its applicability in settings characterised by a lack of centralised control, an underlying network restricting communication, and a topology not wholly known to the individual agents^1,2^. Notable examples are sensor networks, ad-hoc networks, and embedded wireless systems. These are collections of sensing or computing devices equipped with low-range communication capability and deployed within an environment to perform a given task. Other distributed systems are social networks, consisting of individuals who hold opinions and share them with their peers. Distributed averaging is an instance of distributed computation aimed at calculating the global average of a set of values and represents a central task in many networked systems applications. For instance, in clock synchronisation, agents equipped with a local clock communicate to achieve a shared sense of time^3^. In flocking and formation control, on the other hand, moving agents interact with their neighbours to match their direction and velocity^4^. More applications are found in mobile robotic networks^5^, space systems architectures^6^, distributed signal processing and detection^7^, data fusion in sensor networks^8^, load balancing^9^, cooperative coordination and control^10^, and rumour spreading in social networks^11^. Averaging schemes can be applied to both vectors and scalars, and adapted to compute other linear functions and more general calculations^12^. For instance, they have been employed to determine extreme values^13^, estimate network size^14^, and perform distributed summing on networks^15^.

### Gossip algorithms

Distributed averaging algorithms are classified based on the nature of coordination into average consensus (or synchronous) and gossip (or asynchronous) algorithms. Consensus protocols update the values of all nodes simultaneously at discrete time points^4^ and cannot be regarded as truly distributed as they rely on the availability of a global clock shared among all agents^16^. In gossip schemes, on the other hand, nodes periodically communicate with one or few randomly chosen neighbours and independently of each other^17^. Gossip protocols lack the synchronisation requirement, unrealistic for most applications, and can address heterogeneous agents, as well as directional, delayed or failed communication within the same framework^16^. They are particularly attractive for wireless networks because they do not require any specialised routing, do not have a bottleneck or single point of failure, and are robust to changing topology and unreliable network conditions^7^. However, gossip protocols suffer from limited efficiency: nodes can receive the same message several times, so much of their bandwidth is consumed by redundant information^18^. Moreover, they are considerably more challenging to characterise, reproduce and debug due to the added randomness of neighbour selection^19^.

### Performance analysis

The performance analysis of averaging algorithms evaluates the communication required to converge to a sufficiently accurate estimate. It is particularly important in networked systems^12^, which are typically constrained in computation, communication and energy requirements^20^. Previous studies on the performance of gossip algorithms have focused on deriving theoretical performance guarantees in various graph types^21,22^. These results have informed the design of faster and more robust gossip algorithms^23^ tailored to specific structures such as geometric random graphs^24^, ring networks^25^, and 1-dimensional lattices^19^.Topological approaches to performance analysis investigate how the arrangement of nodes and edges in the communication network affects the convergence rate, so that faster convergence can be engineered by acting on the underlying network rather than on the averaging algorithm. Several studies deployed this approach on consensus algorithms, where more performing graphs were obtained by maximising the algebraic connectivity^26,27^. However, similar studies on gossip algorithms are still lacking.

### Topological approaches

Other classes of distributed algorithms have benefited from a topological characterisation, including distributed mutual exclusion algorithms for controlling concurrent access to shared resources^28^. Topology-informed routing algorithms have been proposed to reduce energy consumption and prolong network lifetime^29^. Concurrently, network topology optimisation strategies have been deployed to ensure uniformly distributed traffic and more efficient use of resources^30^. Graph theoretical approaches are well-established in the study of network robustness, relevant to computer networks and power grids. Measures of network robustness deriving from graph metrics are increasingly adopted in the design, development, deployment and operation of telecommunication systems^31^. Such metrics enable identifying vulnerable nodes, investigating network attack mechanisms, and planning defensive strategies^32^. The analysis of real communication systems identified typical graph properties and informed the design of synthetic test beds for power grids^33^. Moreover, graph metrics computed on control flow graphs of applications enabled malware detection in Internet of Things systems^34^. Lossy graph compression schemes preserving graph properties have been developed for high-performance graph processing, storage, and analytics^35^. Topological approaches in machine learning generally concern mapping high-dimensional data to a low-dimensional space to facilitate analysis and visualisation^36^. However, recent work highlighted the effect of complex topological features found in biophysical systems on task performance, applicable to the design of bio-inspired artificial neural networks^37^.

### Contribution

This study investigates the effect of topological features on the convergence rate of gossip algorithms in four graph families representative of real-life networks: Erdős–Rényi^38^ (ER), small world^39^ (SW), scale-free^40^ (SF), and geometric random^41^ (GR) graphs. It focuses on sparse graphs, in which the number of effective connections is much lower than the number of possible connections. These graphs are characterised by communication constraints induced by the topology that limit the algorithm performance. Simulations are deployed to evaluate the topological limiting factors, the most predictive graph metrics, and the most efficient random algorithm for each graph family and for all graphs. A regression model built on network metrics predicts the convergence rate with high accuracy, confirming that topological features determine performance. Local metrics, calculated on each node’s neighbourhood, are almost as predictive as global metrics, calculated on the whole graph, but require significantly fewer computational resources and can be fully parallelised. Notably, averages of local metrics alone retain a high explanatory power. It is suggested that nodes compute local metrics and estimate their mean value by distributed averaging together with the global average of the measured quantities. Nodes then employ estimates of average local metrics to make predictions of the graph convergence rate and the time taken to achieve the desired level of accuracy so that they use their estimate only when confident of their quality. An implementation of this approach confirms that most nodes are able to make prompt and accurate predictions in all considered graph families. The main contributions of this article are as follows:

- To provide a model of the relationship between graph density and convergence rate in different graph families and offer insights on the effect of clustering and rewiring on performance;
- To identify individual graph metrics that are highly predictive for convergence rate and suggest general topological properties associated with high performance;
- To confirm the predictive capabilities of structural features and demonstrate the validity of the topological approach;
- To identify the set of local metrics that minimises computational cost while retaining high explanatory value, and to propose a novel approach to estimate the convergence rate in a distributed manner;
- To offer guidelines for the design, maintenance and improvement of resource-efficient topologies.

## Background and related work

### Distributed averaging

This section reviews mathematical preliminaries of graph theory relevant to the remainder of the article and defines the problem of distributed averaging. Then, it presents the averaging gossip protocol and the asynchronous time model in their most frequently adopted formulation.

#### Network topology

Communication constraints in network systems can be conveniently modelled by a graph $G = (V, E)$, where *V* is the vertex set of *n* nodes $v_i$, with $i \in I$ = {1, ..., *n*} and $n \in \mathbb {N}$, and *E* is the edge set $E \subseteq V \times V$ of the pairs $e_{ij} = (v_{i}, v_{j})$, so that there is an edge between nodes $v_i$ and $v_j$ iff $(v_{i},v_{j}) \in E$. The graph *G* is connected iff a path connecting $v_i$, and $v_j$ exists $\forall$ $i,j \in I$. *G* is simple if it is unweighted, undirected, without loops and multiple edges, meaning that the pairs $e_{ij} \in E$, with $i \ne j$, are unique, unordered, and are not assigned a weight. *G* is complete if it is fully-connected, i.e. $E = V \times V$. The neighbour set of node $v_{i}$ is denoted by $\Omega _{i} = \{v_{j} : (v_{i}, v_{j}) \in E\}$, while the degree of $v_{i}$ is the cardinality of the set, denoted by $deg(v_{i})$. The average degree of *G* is defined as the arithmetic average of the degree of its nodes, i.e. $deg_{avg} = \frac{1}{n}\sum _{v \in V} deg(v)$. The adjacency matrix ${\textbf {A}}(G)$ associated with *G* is the $n \times n$ matrix [$a_{ij}$] such that $a_{ij}$ is equal to 1 $\forall (v_{i}, v_{j}) \in E$ and 0 otherwise. In simple graphs, ${\textbf {A}}(G)$ is symmetric and has all zeroes on its main diagonal. The degree matrix ${\textbf {D}}(G)$ is the diagonal $n \times n$ matrix [$d_{ij}$] such that $d_{ii}$ is equal to $deg(v_{i})$. The Laplacian associated with graph *G* is defined as ${\textbf {L}}(G) = {\textbf {D}}(G) - {\textbf {A}}(G)$. ${\textbf {L}}(G)$ has all non-negative eigenvalues in undirected, unweighted graphs.

#### Asymptotic consensus

Let $x_{i}$ denote the value of node $v_i$ representing an opinion, a measurement, or a state. Then, the nodes $v_i$ and $v_j$ are said to agree in a network iff $x_{i} = x_{j}$, while all nodes in *G* have reached a consensus iff $x_{i} = x_{j} \; \forall i,j \in I$. The vector ${\textbf {x}}(0) = (x_{1}(0), \ldots , x_{n}(0))^{T}$ denotes the initial state of the system, so that the $i^{th}$ component of ${\textbf {x}}(0)$ is the initial value at node $v_{i}$. The vector ${\textbf {x}}(t)$ denotes the vector of the nodes values at time *t*, while ${\textbf {x}}(k)$ represents the discrete counterpart at time-slot *k*. The system reaches asymptotic consensus if all nodes asymptotically converge to the same value, i.e. there exists $x^*$ such that

$$\lim _{t \rightarrow +\infty } x_i(t) = x^* , \forall i \in I.$$

In distributed averaging, the goal is for $x^*$ to be equal to the average of the initial values $x_{avg}$, computed as $\frac{1}{n}\sum _{i = 1}^{n}x_i(0)$.

#### Gossip protocol

The most adopted formulation of the averaging gossip protocol prescribes that a node $v_i$ randomly selects one of its neighbours $v_j$ for interaction. Then, the two nodes exchange their current values and perform local averaging, i.e. updates their value as

$$x_i (k+1) = x_j(k+1) = \frac{x_i(k) + x_j(k)}{2}.$$

The algorithm is defined by the $n \times n$ probability matrix ${\textbf {P}} = [p_{ij}]$, prescribing the probability $p_{ij}$ that the node $v_i$ selects node $v_j$ for interaction. ${\textbf {P}}$ is a stochastic matrix, i.e. its rows sum to 1. Its largest eigenvalue equals 1, while all others are strictly less than 1. Due to the constraints of only interacting with neighbours, $p_{ij} > 0$ if $(v_i,v_j) \in E$. Each interaction is characterised by the $n \times n$ matrix of the averaging weights ${\textbf {W}} = [w_{ij}]$, so that the vector of values is updated as

$${\textbf {x}}(k+1) = {\textbf {W}}(k){\textbf {x}}(k).$$

The interaction between $v_i$ and $v_j$ has weight matrix ${\textbf {W}}_{ij}$ with elements equal to $\frac{1}{2}$ at $w_{ii}$, $w_{ij}$, $w_{jj}$, $w_{ji}$, equal to 1 at $w_{kk}$, with $k \ne i,j$, and 0 otherwise. This is equivalent to setting the values of nodes $v_{i}$ and $v_{j}$ to the average of their current values, leaving the others unchanged. The operation solely relies on the current values of the node and its neighbours, meaning that the process has no memory of the previously computed estimates. ${\textbf {W}}$ is a symmetric doubly stochastic matrix, i.e. all rows and columns sum to 1, and has non-negative real eigenvalues $\lambda$, so that 1 = $\lambda _1({\textbf {W}})$ $\ge$ $\lambda _2({\textbf {W}})$ $\ge$ .. $\ge$ $\lambda _n({\textbf {W}})$ $\ge$ 0. ${\textbf {W}}$ is also a projection matrix, i.e. ${\textbf {W}}^2 = {\textbf {W}}$, because averaging the same pair of nodes a second time does not change the vector ${\textbf {x}}$. In a gossip algorithm, the matrix ${\textbf {W}}$ changes over time as different pairs interact at different times. The averaging process is thus defined by the sequence of averaging matrices, which can be interpreted as the realisation of a stochastic process $\{{\textbf {W}}(t)\}_{t\ge 0}$.

#### Asynchronous time model

**Figure 1:**
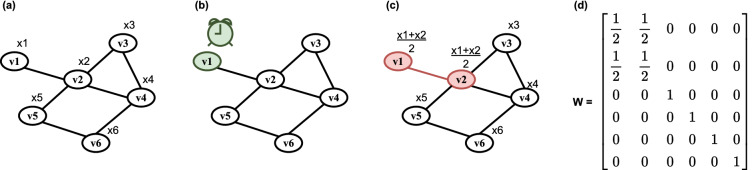
(**a**) Gossip averaging with asynchronous time model performed on a graph, where each node $v_i$ is assigned a value $x_i$. (**b**) At a time $T_{k}$, one of the clocks ticks to signify that the corresponding node ($v_1$) will perform an averaging scheme. (**c**) The node selects a neighbour ($v_2$), and both nodes replace their values with the average of the two. (**d**) This is equivalent to multiplying the vector of all values ${\textbf {x}}$ by the weight matrix ${\textbf {W}}$.

In the asynchronous time model for distributed averaging, outlined in Fig. 1, only one node communicates with others at a given time, in contrast to the synchronous model, where time is commonly slotted across all nodes, and all nodes are updated simultaneously^1^. The asynchronous model marks the passing of time with clocks assigned to each node and ticking at the times of a rate 1 Poisson process so that the time between two consecutive ticks is a rate 1 exponential random variable. This is equivalent to a global clock ticking at times $T_{k}$, with $k \ge 1$, at a rate *n* Poisson process, where inter-tick times ${T_{k} - T_{k-1}}$ are rate *n* exponentials^2^. When a clock ticks, the corresponding node selects a neighbour and performs local averaging. A node $v_{i}$ interacts with node $v_{j}$ at time slot *k* with probability $\frac{p_{ij}}{n}$, which is the joint probability that its clock ticked at time slot *k* (*p* = $\frac{1}{n}$) and that that it selects node $v_{j}$ for interaction (*p* = $p_{ij}$). The vector of estimates ${\textbf {x}}$ remains constant in the interval $[T_{k-1}, T_{k}) \; \forall k$ because it can only change when a clock ticks. Thus, it is convenient to discretise time according to clock ticks and track time in terms of the number of clock ticks. Alternatively, time can be measured in units of absolute time, considering that, on average, there are *n* ticks per unit of time. If the clocks tick at times of a rate *q* Poisson process, $n \; q$ interactions take place per unit of time, about *q* per node.

### Convergence of gossip algorithms

This section presents necessary and sufficient conditions for convergence to the global average, defines the most frequently adopted convergence metrics, and reviews some relevant convergence results on complete and arbitrarily connected graphs.

#### Convergence conditions

An averaging algorithm is said to converge almost surely to the global average $x_{avg}$ if

$$\mathbb {P}\left( \lim _{t\rightarrow \infty } {\textbf {x}}(t) - x_{avg}{} {\textbf {1}} = 0\right) = 1,$$

where ${\textbf {1}}$ is the $n \times 1$ vector of all 1s. Necessary conditions for convergence state that the computation must preserve the global average, i.e. ${\textbf {1}}^TW(t) = {\textbf {1}}^T$, and that the vector of averages must be a fixed point of the iteration. i.e. $W(t){\textbf {1}} = {\textbf {1}}$^1^. Both conditions are always satisfied in gossip algorithms as **W**(t) is a doubly stochastic matrix. These two conservation properties, together with contraction and connectivity properties, represent sufficient conditions for convergence when the corresponding random process $\{{\textbf {W}}(t)\}_{t\ge 0}$ is stationary and ergodic, which is generally satisfied by most network models^42^. The contraction condition states that $\Vert {\textbf {W}}(t)\Vert _2 \le 1$, where $\Vert .\Vert _2$ is the spectral norm of the matrix and is satisfied in gossip averaging algorithms for each ${\textbf {W}}(t)$. The connectivity condition requires the network to be jointly connected, meaning that nodes can be isolated at times but have to eventually connect to the network, and it is always satisfied in connected graphs. It follows that gossip algorithms generally converge to the global average without any coordination required as long as the underlying communication graph is connected.

#### Convergence metrics

The performance of distributed averaging algorithms is generally intended as the resources required to achieve a sufficiently accurate estimate, such as time, messages sent, and bandwidth. Let ${\textbf {err}}(k)$ denote the error vector of the estimates at time slot *k*^42^

$$ {\textbf {err}}(k) = {\textbf {x}}(k) - x_{avg}{} {\textbf {1}} $$

and $err_n(k)$ the error norm normalised by the initial values

$$ err_n(k) = \frac{\Vert {\textbf {x}}(k) - x_{avg}{} {\textbf {1}} \Vert }{\Vert {\textbf {x}}(0) \Vert } = \frac{\Vert {\textbf {err}}(k) \Vert }{\Vert {\textbf {x}}(0) \Vert }$$

where $\Vert . \Vert$ is the $l_{2}$ norm of the vector. If the algorithm converges, the error $err_n(k)$ becomes arbitrarily small when the number of iterations *k* is taken large enough. Notably, $err_n(k)$ is a non-increasing monotonic function of *k* because the distance of a vector ${\textbf {x}}(k)$ from the vector of averages $x_{avg}{} {\textbf {1}}$ can only decrease after averaging two of its values. Let ${\textbf {err}}(k+1)$ be the error vector at time slot $k+1$ after an averaging scheme involving nodes $v_i$ and $v_j$. Then it can be shown that:

$$ \Vert {\textbf {err}}(k+1) \Vert ^2= \sum _{l = 1}^{n}(x_l(k+1) - x_{avg})^2 = \sum _{l = 1}^{n}(x_l(k) - x_{avg})^2 - \frac{(x_i(k) - x_j(k))^2}{2} = \Vert {\textbf {err}}(k) \Vert ^2 - \frac{(x_i(k) - x_j(k))^2}{2}.$$

Thus, any pairwise interaction reduces the error by a quantity that depends on the distance between their values. The fastest averaging gossip algorithm is one that always selects the pair of nodes holding the most distant values at any given time.

Another established theoretical measure of convergence is the *e-averaging time*, denoted by $T_{avg}$, which is the earliest time at which the vector ${\textbf {x}}(k)$ is within *e* of the vector $x_{avg}{} {\textbf {1}}$ with a probability greater than $1 - e$ regardless of the initial values ${\textbf {x}}(0)$. Formally:

$$T_{avg}(e,P) = \sup _{{\textbf {x}}(0)} \inf _{k=0,1,..} \{k: \mathbb {P}(err_n(k) \ge e) \le e \}$$

for any $0< e < 1$^1^. Then, a system is said to *e-converge* if ${\textbf {x}}$ gets within *e* of $x_{avg}{} {\textbf {1}}$, i.e. $err_n \le e$. This probabilistic notion is convenient because it controls both error and probability with the same parameter *e*. However, it may be difficult to evaluate numerically as it requires large samples and multiple experiments. It is generally expressed in terms of the number of clock ticks, although absolute times can be easily retrieved by dividing the number of ticks by the average ticks per unit of time.

Other performance metrics assess the cost of the algorithm up to time *t* for every realisation of the network and algorithm, independently of the initial values and under the assumption that the convergence conditions are satisfied. Let *C*(*t*) be the total cost of the algorithm (in terms of time, number of iterations, power, energy, etc.) guaranteed to converge, and let

$$C_c = \lim _{t\rightarrow \infty } \frac{C(t)}{- log\Vert {\textbf {err}}(t)\Vert _p},$$

where $\Vert . \Vert _p$ is any *p*-norm, with $p \in [1,\infty$). If the limit exists, $C_c$ is the consensus cost of the algorithm. In the particular case of cost equal to time, i.e. $C(t) = t$, if the limit

$$T_c = \lim _{t\rightarrow \infty } \frac{t}{- log\Vert {\textbf {err}}(t)\Vert _p}$$

exists, $T_c$ is the consensus time of the algorithm. It was shown that if the sequence of averaging matrices $\{{\textbf {W}}(t)\}_{t\ge 0}$ is stationary and ergodic, generally satisfied in most networks models, the two limits exist and $C_c = \mathbb {E}[C(1)]T_c$^42^.

**Figure 2:**
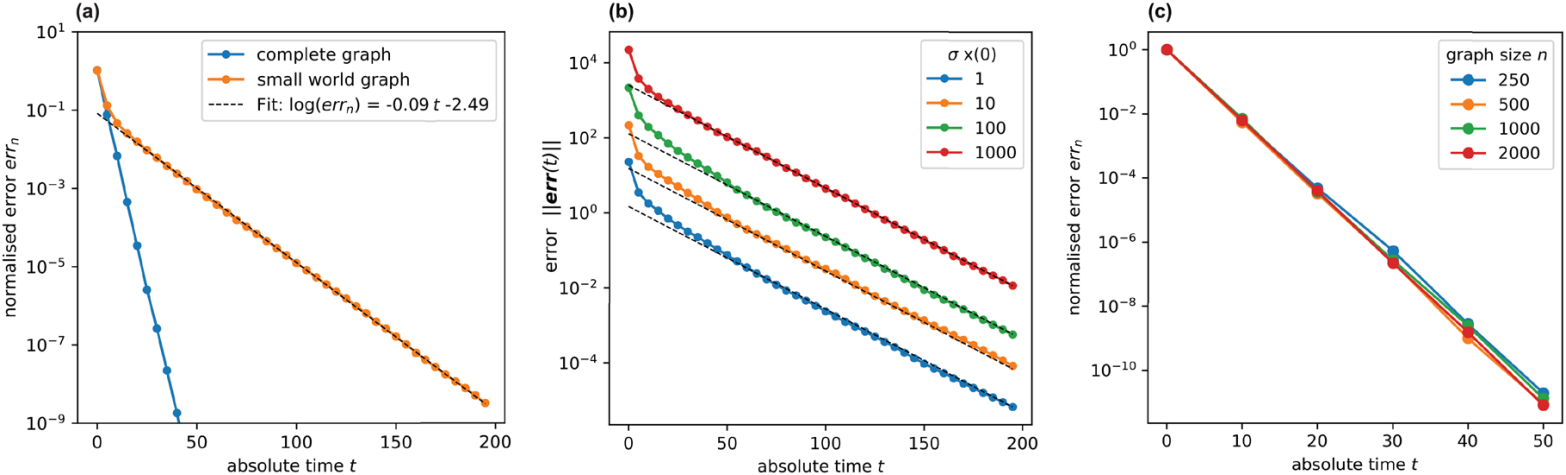
(**a**) In a SW graph with size $n = 1000$ and average degree $deg_{avg} = 20$, after a faster transient phase, the logarithm of the normalised error decreases linearly but at a slower rate than the complete graph of the same size. The convergence rate can be estimated as the angular coefficient of the regression line of the normalised error with respect to time. (**b**) In graphs where nodes are assigned values from a Gaussian random distribution with $\mu = 0$ and $\sigma$ varying in [0, 1000], the angular coefficient of the regression line is constant, i.e. the convergence rate is independent of the initial values. (**c**) The convergence rate in complete graphs is independent of the graph size *n*.

Numerical simulations of gossip protocols have shown that the logarithm of the error $err_n(k)$ decreases linearly after a faster transient phase (Fig. 2a). Moreover, theoretical results on the limit existence suggest that the decreasing rate is deterministic and independent of the initial measurements ${\textbf {x}}(0)$ (Fig. 2b). Hence, a contraction rate $C_r$ can be defined as the angular coefficient of the linear stationary regime and used to characterise the algorithm performance^42^.

#### Convergence in complete graphs

The convergence rate of gossip algorithms intuitively depends on the density of the graph, defined as the ratio between the number of edges and the maximum number of possible edges^43^. Denser graphs can spread information more efficiently and require fewer messages to achieve the desired accuracy level. In contrast, sparser graphs display topology-induced communication constraints limiting the converge rate. It follows that complete graphs maximise the convergence rate for graphs of a given size *n*. It was shown that, in these graphs, the number of interactions required to *e*-converge is bounded by $$\Theta (n \; log \; e^{-1})$$, with $$\Theta $$ indicating an upper and lower bound (or tight bound) for the convergence function^44^. For nodes interacting at a rate *q* Poisson process, $$n \; q$$ interactions take place per unit of time (*q* per node), so the absolute time to *e*-converge is bound by $$\Theta (\frac{1}{q} \; log \; e^{-1})$$. Thus, in the representative case of $$q = 1$$, the time to *e*-converge is bound by $$\Theta (log \; e^{-1})$$ and is independent of the graph size (Fig. 2c). It was also observed that certain types of well-connected graphs *e*-converge with the same number of messages as the complete graph. However, topologies relevant to ad-hoc sensor networks, such as grids and geometric random graphs, *e*-converge with $$\Theta $$ ($$n^2$$ *log* $$e^{-1})$$, which is comparable to messages necessary for each node to flood its value to all other nodes^42^.

#### Convergence in connected graphs

**Figure 3:**
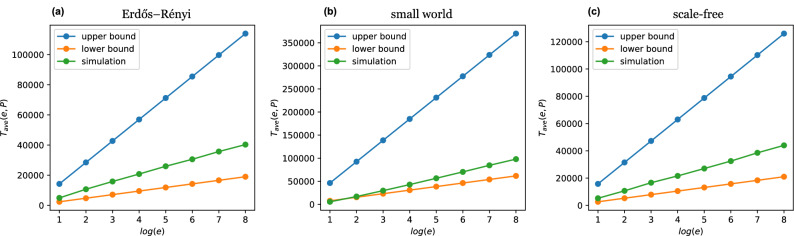
Upper and lower bounds of the *e-averaging time*, $T_{avg}$, defined by Boyd et al.^1^ and the $T_{avg}$ calculated through simulations in (**a**) Erdős-Rényi, (**b**) small world and (c) scale-free graphs with $n = 1000$ and $deg_{avg} = 20$.

Boyd et al.^1^ showed that for any connected graph, the convergence time of gossip algorithms is closely related to the mixing time of a Markov chain defined on the graph topology and provided a tight characterisation of the averaging time, shown in Fig. 3 and defined as follows:

$$ \frac{0.5 \, log \, e^{-1}}{log \, (\lambda _2 \, (\bar{{\textbf {W}}}))^{-1}} \le T_{avg}(e,{\textbf {P}}) \le \frac{3 \, log \, e^{-1}}{log \, (\lambda _2\, (\bar{{\textbf {W}}}))^{-1}}$$

where $\bar{{\textbf {W}}}$ denotes the expected weight matrix $\mathbb {E}[W] =\frac{1}{n}\sum _{i,j} p_{ij}{} {\textbf {W}}_{ij}$ and $\lambda _2$ is its second-largest eigenvalue taking values in the interval (0, 1). $\bar{{\textbf {W}}}$ is most commonly found in the form:

$$ \bar{{\textbf {W}}} = {\textbf {I}} - \frac{1}{2n}{} {\textbf {D}} + \frac{{\textbf {P}} + {\textbf {P}}^T}{2n},$$

where ${\textbf {D}}$ is the diagonal matrix with entries

$$ D_i = \sum _{j=1}^{n}[p_{ij} + p_{ji}].$$

Thus, the averaging time is a monotonically increasing function of the second-largest eigenvalue of $\bar{{\textbf {W}}}$. The problem of finding the fastest averaging algorithm can then be cast as the optimisation problem of finding the probability matrix ${\textbf {P}}$ that satisfies all constraints and minimises $\lambda _2(\bar{{\textbf {W}}})$^1^. Boyd et al. observed that the optimisation problem could be solved efficiently and proposed an algorithm to perform the optimisation distributively^2^.

### Network families

**Figure 4:**
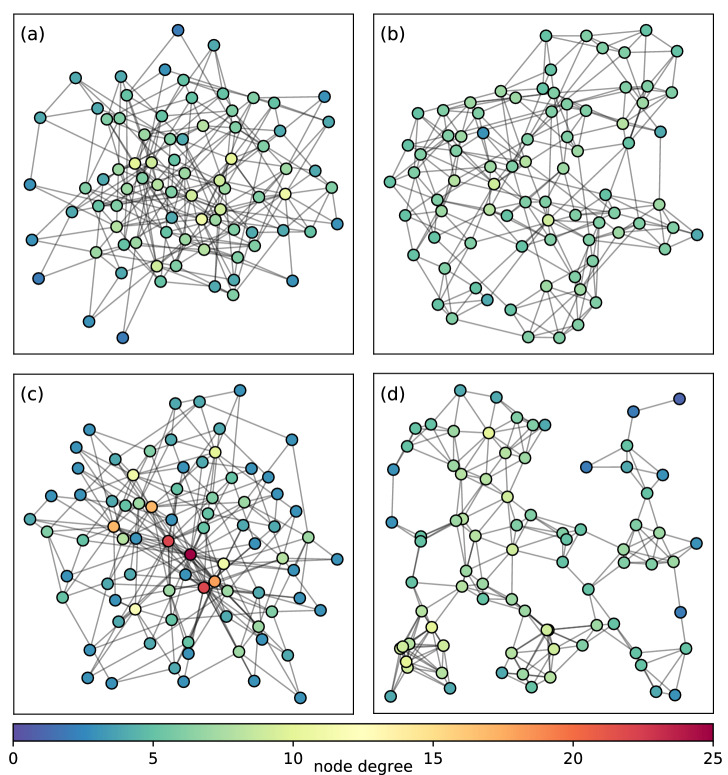
Examples of graphs for the four considered graph families: (**a**) Erdős-Rényi (**b**) Watts–Strogatz small world (**c**) Barabasi–Alber scale-free (**d**) geometric random graphs. All graphs have network size $n = 80$ and average degree $deg_{avg}\approx 6$. Nodes are coloured based on their degree.

This section describes the four considered graph families, modelled after real-world networks. It outlines the corresponding generative models and provides the parameter configurations for generating connected graphs. Statistical and topological properties are briefly discussed for each family. A graphical representation of a graph for each family is shown in Fig. 4.

#### Erdős-Rényi networks

Erdős-Rényi networks are random graphs where each pair of nodes is connected with probability *p*, i.e. $\mathbb {P}((v_i,v_j) \in E) = p$ $\forall i,j \in I, i\ne j$^38^. This is equivalent to selecting a graph uniformly and at random from the set of all graphs with fixed size *n* and number of edges |*E*|. The parameters of the generative model are the number of nodes *n* and the probability of edge formation *p*. The graphs are obtained by adding each possible edge to the edge set *E* with probability *p*. The degree distribution is binomial and can be approximated by a Poisson peaking around the expected average degree $deg_{avg} = p\;(n - 1)$. Most nodes have degrees in the narrow vicinity of $deg_{avg}$, so they are statistically homogeneous. Notably, the degree distribution does not depend on the network size but solely on $deg_{avg}$. Several other properties solely depend on the $deg_{avg}$, so it is considered the most defining property of this graph family. The expected number of edges in a ER graph is equal to the number of all possible edges multiplied by the probability of each edge, i.e. $|E| = \frac{p\,n\,(n-1)}{2}$. The graph will almost surely be connected if the $p > \frac{log\,(n)}{n}$^38^.

#### Watts–Strogatz small world networks

Small world networks are characterised by an average path length that depends logarithmically on the graph size *n* rather than polynomially, as in regular lattices. Communication is efficient because distances are orders of magnitude smaller than the system size. In real networks, the small world property is generally coupled with a high average clustering coefficient. The Watts–Strogatz model (also denoted as the small world model) interpolates between an ER graph, with low average path length and low clustering coefficient, and a regular lattice, having opposite properties. SW graphs are defined by the number of neighbours per node *h*, equivalent to the average degree $deg_{avg}$, and the probability of randomly rewiring each edge $p_r$. The degree distribution is symmetric and centred on $deg_{avg}$. The generating algorithm constructs a regular ring lattice, i.e. a ring of *n* nodes, each connected to *h* neighbours (or $h - 1$ if *h* is odd) with $\frac{h}{2}$ on each side. Then, it takes every edge and rewires it to a randomly chosen one with probability $p_r$^39^. Loops and multiple edges are prevented in the rewiring procedure, and the number of links is kept constant, regardless of $p_r$. While the initial ring lattice is connected, the rewired graph not necessarily is. Note that the $p_r$ parameter controls the “randomness” of the graph: $p_r = 0$ corresponds to the original highly structured ring lattice, while $p_r = 1$ results in an ER random network. The SW model overcomes the shortcomings of the ER model, which cannot generate local clustering and triadic closures. However, it produces an unrealistic degree distribution and implies a fixed number of nodes, so it cannot be used to model growth.

#### Barabasi–Albert scale-free networks

Scale-free networks have a degree distribution that follows a power-law so that the fraction of nodes having a given degree $\mathbb {P}(deg)$ is asymptotically approximated by $deg^{-\gamma }$. The degree of a randomly selected node can be arbitrarily large, hence the lack of scale, unlike ER and SW graphs, where the degree varies within a narrow range. The Barabasi–Albert (BA) model to reproduce scale-free properties uses a preferential attachment mechanism, where a graph is grown by attaching each new node to *m* existing nodes with probability proportionate to their degree^40^. The only parameter of the model is the number of links *m* created for each new node, which determines the average degree of the graph. If the initial graph is connected, the resulting graph is necessarily connected too. The Holme and Kim algorithm implements the BA model with an extra step, where forming a random edge is followed by connecting with one of its neighbours with probability $p_c$^45^. This extension to the BA model generates scale-free graphs with tunable average clustering.

#### Geometric random networks

Geometric random graphs are constructed by placing *n* nodes uniformly and independently in a metric space. Each pair of nodes is connected if their Euclidean distance is smaller than the selected radius *r*^46^. Thus, the two characteristic parameters for this family are the size of the graph *n* and the radius *r*. The average degree in the unit cube is approximated by $\pi r^2 n$, so the radius of a graph with a desired average degree can be computed as $\sqrt{\frac{deg_{avg}}{\pi \; n}}$^47^. In a 2-dimensional space, the graph will almost surely be connected if $r > \sqrt{\frac{log(n)}{\pi \; n}}$^41^. It was shown that, in order to have good connectivity while minimising interference, the radius *r*(*n*) has to scale like $\Theta (\sqrt{\frac{log(n)}{n}})$^48^.

## Proposed approach

The proposed approach is articulated as follows. The *Graph topologies* section defines the properties of the graphs considered in the investigation and details the procedure to generate them. It also motivates the choice of the four graph families and briefly compares their structural and statistical properties. The *Graph metrics* section presents the metrics chosen to quantify global and local properties of the graphs. It provides the definition, the computation complexity, the significance and primary usage for each metric. The *Gossip schemes* section defines the neighbour selection criteria for the four gossiping algorithms compared in the investigation. The *Gossip implementation* section provides a technical overview of the tool implemented to simulate distributed averaging and explains how to calculate the convergence rate. Finally, the *Experimental design* section outlines the numerical experiments and data analysis conducted within the study.

### Graph topologies

The study investigates four network families representative of real-life networks. ER and GR graphs are benchmarks for ad-hoc wireless sensor networks because they can model unreliable communication across the network and low-range interactions among geolocalised devices. SF and SW networks are more suitable for describing different aspects of social networks, such as the presence of hubs and the tendency of connected nodes to have shared connections. The investigation only considers time-invariant topologies to limit the randomness of the experiments to node interactions. Consequently, the considered graphs are connected to guarantee the convergence of the gossip algorithm. Graphs are also assumed to be undirected and unweighted. The investigation focuses on sparse graphs, here intended as graphs with a density below 0.25, meaning that less than 1/4 of all possible connections are realised in the network. These graphs display topology-induced communication constraints and allow investigating the limiting factors of convergence rate.

The study of the effect of the model parameters on performance generates graphs by fully exploring the parameter space. ER graphs are obtained by varying *n* in [100, 1000] and *p* in the interval $[\frac{log(n)}{n}, 1]$, where $p = 1$ corresponds to the complete graph of size *n*. SW graphs are generated using the Watts–Strogatz model by varying *n* in [100, 1000], *h* in [2, *n*], and $p_r$ in [0,1] in steps of 0.1. SF graphs are generated by varying *n* in [100, 1000], *m* in [1, *n*], and $p_c$ in [0,1] in steps of 0.1. SF graphs with $p_c$ = 0 are equivalent to those generated by the BA model and will be referred to as BA graphs. Geometric graphs are generated by varying *n* in [100, 1000] and taking *r* so that it is a multiple of $\sqrt{\frac{log(n)}{n}}$ greater than $\sqrt{\frac{log(n)}{\pi \; n}}$. Simple and multiple regression analyses of graph metrics are conducted on a set of over 12000 graphs (of which $\approx 1600$ ER, $\approx 1600$ GR, $\approx 4400$ SW, $\approx 4400$ SF) having sizes uniformly drawn in the interval [200, 1000] and average degree ranging from the minimum value generating connected graphs up to 60, above which maximum convergence is almost always achieved.

### Graph metrics

The study identifies a set of metrics that quantify global and local properties of the graph and capture a variety of topological features^49^ contributing to the algorithm convergence rate. This section provides, for each selected metric, the formal definition and the complexity of its computation, as well as its significance and related applications motivating its choice. Metrics are here classified into global, local and spectral metrics. Global metrics are calculated on the entire graph and are more meaningful for assessing the state of the whole network and comparing different structures. In contrast, local metrics are calculated on each node’s immediate neighbourhood and provide more detailed insight while requiring fewer computing resources^50^. Spectral metrics refer to the extreme eigenvalues associated with the Laplacian and adjacency matrices. The metrics classification adopted in this study is available in Table 1. For metrics calculated on nodes, such as eccentricity, clustering, local efficiency, and all centrality metrics, five summary statistics are chosen to characterise the distribution: average (avg), maximum (max), minimum (min), standard deviation (std) and skewness (skew). Maxima and minima are particularly significant because they identify nodes acting as a limiting factors for algorithm convergence. For instance, if the clustering coefficient negatively correlates with performance, the node with the highest clustering is likely to limit convergence.

#### Global metrics

The selected global metrics assess centrality, eccentricity, distance and efficiency within a graph. Centrality metrics quantify the position and influence of nodes in the network. The degree centrality $C_D$ for a node $v_i$ is the fraction of nodes it connects to, i.e. the degree of $v_i$ normalised by the largest possible degree $n - 1$. The betweenness centrality $C_B$ measures the fraction of shortest paths in the graph passing through node $v_i$, i.e.

$$C_B(v_i) = \sum _{v_j \ne v_i \ne v_k \in V} \frac{ \rho _{v_j,v_k}(v_i)}{ \rho _{v_j,v_k}}$$

where $\rho _{v_j,v_k}$ is the number of the shortest paths from $v_j$ to $v_k$ and $\rho _{v_j,v_k}(v_i)$ the number of those paths passing through $v_i$. The closeness centrality $C_C$ of a node $v_i$ quantifies the average distance from all other nodes and is defined as

$$C_C(v_i) = \frac{n-1}{\sum _{v_j \in V} \delta (v_i,v_j)}$$

where $\delta (v_j,v_k)$ is the shortest-path distance between the nodes and $n-1$ the number of reachable nodes. $C_c$ measures the physical centrality of a node because a more central node is necessarily closer to all other nodes. Efficient algorithms require $O(|V|\times |E|)$ computation steps, $O(|V|^3)$ in the worst case, to calculate the exact betweenness and closeness centralities for the whole graphs by conducting a breadth-first search from each node^51^. The eigenvector centrality $C_E$ estimates the importance of a node based on that of its neighbours. $C_E(v_i)$ is defined as the $i^{th}$ element of the vector ${\textbf {y}}$ that is solution to the equation ${\textbf {A}}{} {\textbf {y}} = \lambda _{max} {\textbf {y}}$, where ${\textbf {A}}$ is the adjacency matrix of the graph and $\lambda _{max}$ the largest eigenvalue associated with the eigenvector of **A**. This eigenvector can be computed by the power iteration method in $O(|V|+|E|)$ time^51^. The eccentricity *ecc* of a node, on the other hand, is a measure of non-centrality defined as the maximum distance from a given node to any other node in the graph. The maximum and minimum eccentricity are the diameter $\delta _{max}$ and the radius $\delta _{min}$^52^:

$$ecc(v_i) = \max _{v_j \in V} \, \delta (v_i,v_j), \;\; \delta _{max} = \max _{v_i, v_j \in V} \delta (v_i,v_j), \;\; \delta _{min} = \min _{v_i\in V} \, \max _{v_j\in V} \, \delta (v_i,v_j)$$

The chosen distance metrics are the average shortest path length $\delta _{avg}$ and the Wiener index $W_i$, respectively defined as the average and the sum of the distance over all pairs of reachable nodes^53^:

$$ \delta _{avg} = \sum _{v_i, v_j \in V} \frac{\delta (v_i,v_j)}{n(n-1)}, \;\; W_i = \frac{1}{2} \sum _{v_i, v_j \in V} \delta (v_i,v_j)$$

The efficiency *eff* of a pair of nodes is defined as the multiplicative inverse of the shortest path distance, i.e. $\textit{eff}(v_i,v_j) = \frac{1}{\delta (v_i,v_j)}$. Then, the global efficiency of a graph $\textit{eff}_G$ is the average efficiency of all pairs of nodes and measures how effectively it exchanges information^54^. Computing eccentricity, global efficiency, average shortest path length and Wiener index has complexity $O(|V|^3)$.

#### Local metrics

Local metrics comprise degree, efficiency and clustering measures. Degree metrics include the degree average, entropy and assortativity. Other degree statistics (max, min, std and skew) are not calculated, as they can be retrieved from the degree centrality by accounting for the graph size. The Shannon entropy of the degree distribution, named degree entropy, is a measure of disorder, complexity and heterogeneity of the graph, as well as of the level of information that it can encode, defined as

$$ \mathscr {H} = - \sum _{i=1}^{n-1}\,p_d(i)\,log \,p_d(i),$$

where $p_d$ is the probability function of the degree *deg*, so that $p_d(i) = \mathbb {P}( deg = i)$. The assortativity correlation coefficient *ac* quantifies the tendency of a node to attach to other nodes with a similar degree and is calculated as the Pearson correlation coefficient of the node degrees at each end of an edge. The local efficiency $\textit{eff}_L$ of a node $v_i$ is the global efficiency of the sub-graph induced by the neighbours of $v_i$. It quantifies the resistance to failure on a small scale because it measures how effectively information is exchanged after removing a node^54^. The clustering coefficient *cl* of a node $v_i$ is defined as the number of triangles passing through that node $T(v_i)$ divided by the number of possible triangles:

$$cl(v_i) = \frac{2T(v_i)}{deg(v_i)(deg(v_i)-1)}$$

The computational complexity of local metrics largely depends on the network density. In complete graphs, calculating the clustering coefficient has complexity $O(|V|^3)$, $O(|V|^2)$ for each node. If nodes have, on average, $\sqrt{n}$ neighbours, global complexity falls to $O(|V|^2)$, while if all nodes have only two neighbours, complexity becomes linear. For very sparse graphs, local metrics can be computed by each node in constant time and in a fully parallel fashion.

#### Spectral metrics

The spectral metrics are the two largest and two smallest eigenvalues of the adjacency matrix ${\textbf {A}}(G)$ and the Laplacian matrix ${\textbf {L}}(G)$, denoted as $\lambda _i$ where *i* is the size ranking. The smallest eigenvalue of ${\textbf {L}}(G)$ is not included because it is always equal to 0 in connected graphs. The second smallest eigenvalue of ${\textbf {L}}(G)$, also called algebraic connectivity, measures the connectivity of the graph and assesses its robustness and synchronisability. The computational complexity of determining the eigenvalues is $O(|V|^3)$ in general but can be significantly smaller if the matrix is sparse.

**Table 1:** Metrics classification.

Global metrics	Centrality metrics	avg, min, max, std, skew of degree centrality
		avg, min, max, std, skew of betweenness centrality
		avg, min, max, std, skew of closeness centrality
		avg, min, max, std, skew of eigenvector centrality
	Eccentricity metrics	avg, min, max, std, skew of eccentricity
	Distance metrics	average shortest path, Wiener index
	Efficiency metrics	average global efficiency
Local metrics	Degree metrics	avg of degree, assortativity correlation, entropy degree
	Clustering metrics	avg, min, max, std, skew of clustering coefficient
	Efficiency metrics	avg, min, max, std, skew of local efficiency
Spectral metrics	Adjacency metrics	$\lambda _1$, $\lambda _2$, $\lambda _{n-1}$, $\lambda _n$ of the adjacency matrix
	Laplacian metrics	$\lambda _1$, $\lambda _2$, $\lambda _{n-1}$ of the Laplacian matrix

### Gossip schemes

The study proposes and compares four gossiping algorithms which differ in probability matrix *P*, i.e. the criteria to choose a neighbour for interaction. This section provides a formal characterisation of each gossip scheme and motivates the choice of the selection criteria. All proposed algorithms converge to the global average because they satisfy sufficient convergence conditions in connected graphs: each node initiates interactions with non-zero probability and selects each possible neighbour with non-zero probability^55^. All gossip schemes are distributed because each node retrieves the necessary information, such as neighbours degrees and the number of shared connections, only by communicating with its immediate neighbours. All algorithms adopt a *push-pull* messaging strategy^56^, where both gossiping nodes share their value, regardless of whom initiated the interaction. It is the default strategy in distributed averaging because the *push* and *pull* schemes, in which a single node communicates its value, cannot guarantee convergence to the global average.

#### Random selection

**Figure 5:**
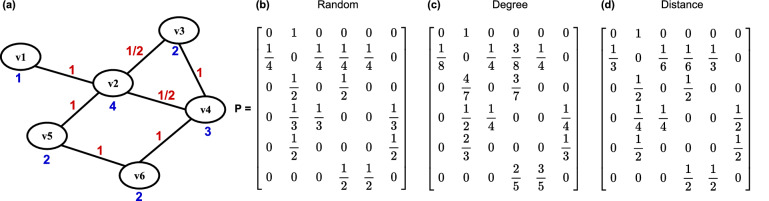
(**a**) Graph topology with node degrees $deg(v_i)$ and edge distances $t_{ij}$ respectively annotated in blue and red. Probability matrix ${\textbf {P}}$ for the corresponding (**b**) random, (**c**) degree and (**d**) distance gossip algorithms.

Each node randomly chooses a neighbour with equal probability. The corresponding probability matrix ${\textbf {P}}$ is [$p_{ij}$] such that

$$p_{ij} = \frac{1}{deg(v_i)} \; \forall v_j \in \Omega _{i}$$

and 0 otherwise, as shown in Fig. 5b. This is equivalent to dividing each row of the adjacency matrix *A* by the degree of the corresponding node. It is the most adopted neighbour criterion and assumes that all neighbours have a comparable influence on the value of a node.

#### Degree selection

Each node preferentially selects more connected neighbours because they are more likely to hold new information, having access to a larger pool of values. The probability of choosing a node is proportional to its degree, so ${\textbf {P}}$ has elements

$$p_{ij} = \frac{deg(v_j)}{\sum _{v \in \Omega _{i}} deg(v)} \; \forall v_j \in \Omega _{i}$$

and 0 otherwise, as seen in Fig. 5c. This scheme uncovers the effect of favouring hubs on performance in heterogeneous topologies like scale-free graphs.

#### Distance selection

Each node preferentially chooses neighbours with fewer shared connections, as they are less likely to propagate redundant information. The selection probability is inversely proportional to the number of shared neighbours and proportional to their distance. Formally, let $s_{ij} = |\{v_k : (v_i, v_k) \in E \wedge (v_k, v_j) \in E\}|$ denote the number of shared neighbours of nodes $v_i$ and $v_j$. The distance between the same nodes is inversely proportional to $s_{ij}$ and can be defined as $t_{ij} = 1/(s_{ij} + 1)$, where $t_{ij} \in (0,1]$ and $t_{ij} = 1$ when the pair has no common neighbours, exemplified in Fig. 5a. The probability matrix ${\textbf {P}}$ has elements

$$p_{ij} = \frac{t_{ij}}{\sum _{v_k \in \Omega _{i}} t_{ik}} \; \forall v_j \in \Omega _{i}$$

and 0 otherwise, as shown in Fig. 5d. Notably, $s_{ij}$ = $s_{ji}$ and consequently $t_{ij}$ = $t_{ji}$ $\forall (v_i,v_j) \in E$, so the distance is a property of the edges rather than the nodes. Graphs generated by randomising regular clustered structures, such as small world graphs, are characterised by a heterogeneous distance profile. Each node has many shared connections with most neighbours but only a few with some neighbours due to rewiring. The effect of randomisation in these structures can be characterised using this selection criterion.

#### Ordered selection

**Figure 6:**
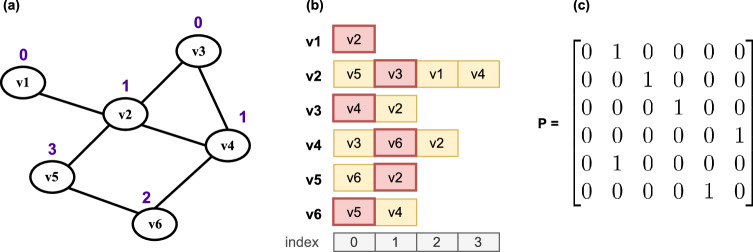
(**a**) Graph topology annotated with the number of interactions $count_i$ initiated by each node. (**b**) List of neighbours for each node in random order. The value of $count_i$ identifies the position in the neighbour list of the next node to contact according to the formula $o = count_i \; \% \; deg(v_i)$. In this implementation, the index of the first element in the lists is 0. (**c**) Probability matrix of order selection for this configuration. In this example, node $v_5$ has 2 neighbours and has interacted 3 times, so $o = 3\%2 = 1$, meaning that the next node to select is at index 1 of the neighbour list, i.e. $v_2$. At the next interaction, $v_5$ will select $v_2$ with probability 1.

Each node chooses its neighbours in a given random order. This strategy maximises the time between interactions with the same node and increases the likelihood that the chosen node holds new information. Unlike other gossip algorithms, the selection step is deterministic. Randomisation only occurs in the beginning when choosing the order to reduce the chance of node pairs synchronising in the network. Let $count_i$ be the number of interactions initiated by node $v_i$, ${\textbf {O}}_i$ the $deg(v_i) \times 1$ vector of the elements in $\Omega _{i}$ taken in a random order, and $o = count_i \; \% \; deg(v_i)$, where $\%$ is the modulo operator. ${\textbf {P}}$ has elements $p_{ij}$ equal to 1 if $v_j$ is the $o^{th}$ element of ${\textbf {O}}_i$ and 0 otherwise. The scheme is exemplified in Fig. 6. In ordered and random selections, nodes select their neighbours an equal number of times. Comparing these strategies sheds light on the effect of randomness on performance.

### Gossip implementation

The study simulates the execution of gossip algorithms to quantify and compare their performance in different graph topologies. The simulator developed within the investigation accepts a graph, a vector, and a gossip criterion as inputs. The graph provides the communication topology for the system, while the vector stores the initial values of the nodes. Values are drawn from a Gaussian distribution with $\mu = 0$ and $\sigma = 1$, although the convergence rate does not depend on the distribution of the initial values. The tool implements an asynchronous time model with interaction rate $q = 1$. At the start of the simulation, each node generates an exponential random variable of rate 1, corresponding to the time the node must wait before initiating an interaction. Each node schedules an interaction event and remains inactive until the waiting time has elapsed. At the time of the event, the node selects one of its neighbours according to the gossip criterion, exchanges values and performs local averaging. Then, it generates a new random waiting time and schedules the following interaction event. The simulator adopts a priority queue to store a representation of events scheduled to happen in the future, and steps in time from one event to another, as no value changes are assumed to occur between consecutive events. The cost of the algorithm is estimated in units of absolute time *t*. Each node is active about once per unit of time, so the number of exchanged messages up to *t* can be retrieved by multiplying *t* by *n*. The convergence rate is calculated as the angular coefficient $C_r$ of the best-fit linear regression line

$$log(err_n(t)) = - C_r \; t + c. $$

**Figure 7:**
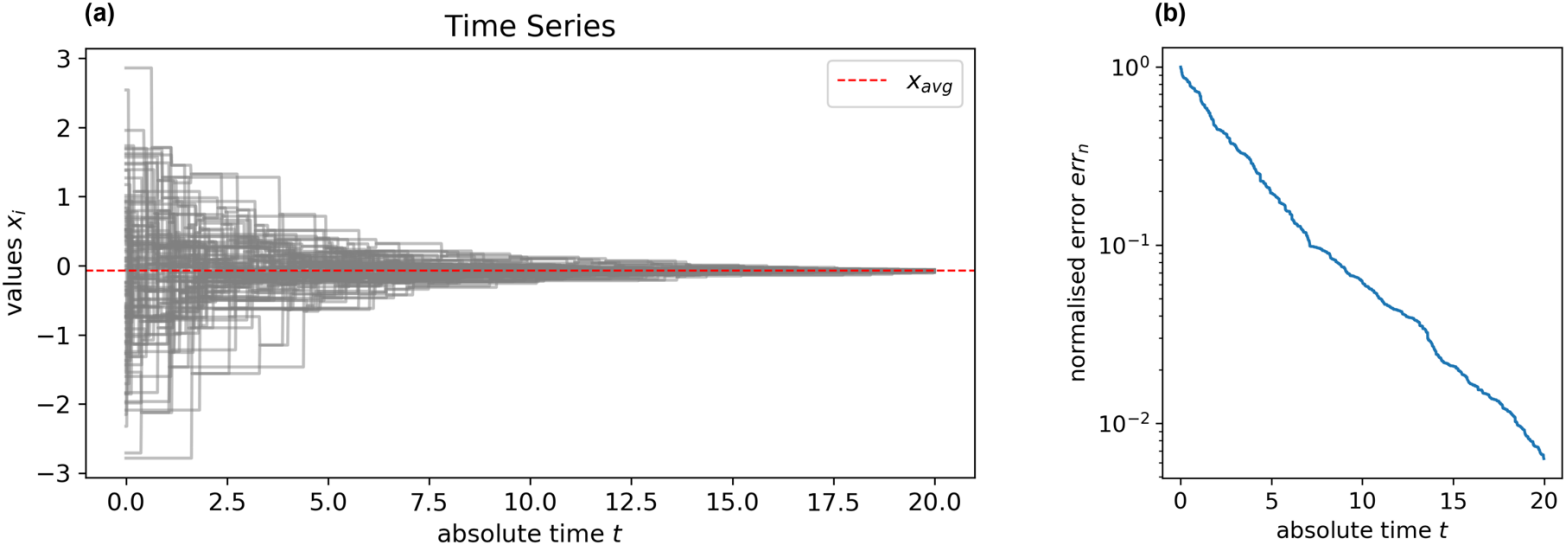
(**a**) Values $x_i$ evolving over time and converging to the average of the initial values $x_{avg}$ in a ER graph with $n = 100$ and $deg_{avg} = 5$ with (**b**) the corresponding normalised error $err_{n}$.

The regression only considers errors computed in the second half of the simulation time to exclude the initial accelerated trend. The simulation time is 50 for ER, SW, and SF graphs and 500 for GR graphs to guarantee that the regression falls in the stationary regime and that error is not affected by the limitations of the computing device. All numerical experiments are executed 100 times. The convergence rate is calculated as the arithmetic average of the rates computed in each simulation run. Figure 7 provides a graphical representation of a time series of values $x_i$ reaching a consensus due by distributed averaging and the corresponding normalised error calculated over time.

### Experimental design

The first simulation set aimed to characterise, within each graph family, the relationship between the parameters of the corresponding generative model and the performance of the random gossip algorithm. Over 1000 graphs are generated per graph family by uniformly sampling the parameter space for connected graphs. Then, the simulator runs the random gossip algorithm on each graph (100 repetitions per graph) and computes the average convergence rate for each combination of parameters. The convergence rate is plotted against each parameter (or functions of them) to identify trends. For instance, $deg_{avg}$ is investigated for ER graphs because it is the product of the two generative parameters *p* and *n*. Then, linear and non-linear regression models are fitted to the data, and the most predictive model is identified for each graph family.

The second simulation set investigates the predictive power of individual graph metrics and groups of metrics over the convergence rate for each graph family and all graphs. In particular, the experiments evaluate the predictive capabilities of local metrics, which can be computed locally by each node and at a low computational cost. The study generates over 12,000 sparse networks and, for each of them, calculates 48 metrics and the average convergence rate of the random gossip algorithm. The effect of each metric on performance is estimated by computing the coefficient and r-squared value of the linear regression model. Then, multi-linear regression analysis is used to evaluate and compare the predictive power of groups of metrics. Several combinations of metrics are compared to identify the set with the fewest metrics (or with the lowest computational cost), retaining high explanatory value. The predictive value of local metrics is investigated further by implementing an extension to the simulator that calculates local metrics and uses them to predict the graph convergence rate at each node. Simulations are deployed to assess the accuracy of nodes predictions in each graph family.

The last simulation set compares the performance of each gossip algorithm in the four graph families. Over 200 graphs are generated per graph family by varying the most predictive parameter for that family over the range that generates connected graphs. The simulator runs the four gossip algorithms on each graph (100 repetitions per graph) and computes the average convergence rate. These values are then plotted and analysed to identify differences in performance induced by topology.

## Results

### Relationship between parameters of the generative model and algorithm performance

Extensive simulations on each graph family investigate the relationship between the parameters of the corresponding model and the performance of the random gossip algorithm. The parameters are found to be highly predictive of the algorithm convergence rate. The study proposes non-linear regression models describing these relationships with high accuracy.

#### ER graphs

Simulations investigating the effect of parameters on performance found that the relationship between the convergence rate and the average degree follows an asymptotic regression model^57^ of the form

$$y = \alpha + \beta \; e^{(- \gamma \; x)}.$$

**Figure 8:**
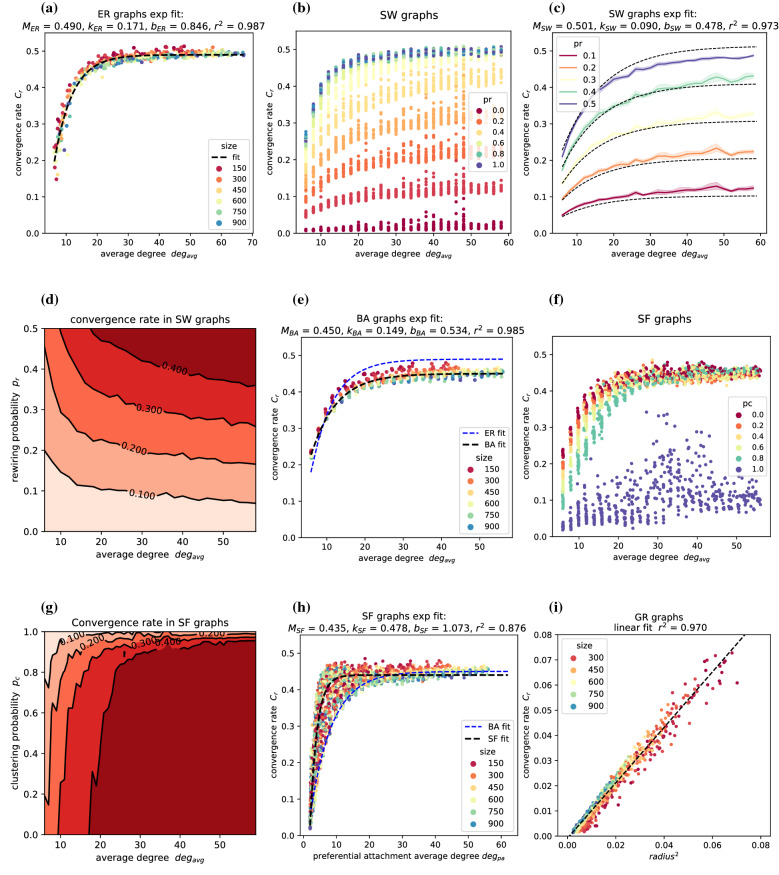
Average degree versus convergence rate and corresponding asymptotic regression model in (**a**) ER graphs, (**b**) SW graphs, (**c**) SW graphs with $p_r <=$ 0.5, (**e**) BA graphs, and (**f**) SF graphs. Contour plots of the two parameters of the generative model for (**d**) SW graphs and (**g**) SF graphs. (**h**) Preferential attachment average degree versus convergence rate in SF graphs. (**i**) Radius squared versus convergence rate in GR graphs. Each convergence value is the average convergence rate obtained over 100 simulation runs.

In ER graphs, the convergence rate $C_{r}$ of the random gossip algorithm increases with $deg_{avg}$ according to

$$C_{r} = M_{ER} - b_{ER} \; e^{(- \; k_{ER} \;\; deg_{avg})},$$

where $M_{ER}$ is the maximum convergence rate, and $k_{ER}$ is the rate at which the maximum rate is approached (Fig. 8a), and $b_{ER}$ is the coefficient of the exponential term, taken with opposite sign to highlight that $M_{ER}$ is an upper bound for the function. The estimated parameters and the 95% bootstrap confidence interval (obtained performing 10000 resamples with replacement) are $M_{ER}$ = 0.490 [0.489, 0.491], $k_{ER}$ = 0.171 [0.158, 0.187], $b_{ER}$ = 0.846 [0.750, 0.979]. Notably, also the relative size of the giant component in ER graphs grows with $deg_{avg}$ according to a similar exponential model and independently of the network size^38^. The model fully characterises the convergence rate (r-squared = 0.987) and estimates the maximum rate $M_{ER}$ at 0.490, close to the rate calculated for complete graphs (0.500). According to this model, as the average degree increases, the convergence rate first steeply increases and then levels off. For instance, 90% of the maximum rate $M_{ER}$ is already achieved at an average degree of 16, while 99%, 99.9% and 99.99% of $M_{ER}$ are respectively reached at $deg_{avg}$ equal to 28, 40 and 52. Consequently, a convergence rate close to that observed in a complete graph can be achieved in a much sparser ER graph. A connected graph of 1000 nodes measures a convergence rate of 0.500, while an ER graph with equal size and an average degree of 25 achieves a convergence rate of 0.480 with only 2.5% of the edges.

#### SW graphs

Simulations results for SW graphs, shown in Fig. 8b, indicate that for $p_r$ below 0.5, $deg_{avg}$ determines the rate according to the same asymptotic model, but the curve is scaled by a factor proportional to the rewiring probability $p_r$ (Fig. 8c):

$$C_{r} = 2 p_r \; (M_{SW} - b_{SW} \; e^{(- \; k_{SW} \;\; deg_{avg})}),$$

where $p_r$ is multiplied by two so the fitted parameter $M_{SW}$ corresponds to the maximum rate attainable for $p_r \le 0.5$. The model describes the data accurately (r-squared = 0.973) and has parameters $M_{SW}$ = 0.501 [0.498, 0.504], $k_{SW}$ = 0.090 [0.086, 0.094], $b_{SW}$ = 0.478 [0.464, 0.491]. At $p_r$ equal to 0.5, almost all nodes have been rewired because each edge has a 0.5 probability of being randomised by each node. For $p_r$ above 0.5, the graph resembles and behaves similarly to an ER graph and can be described by the ER asymptotic model (r-squared = 0.877). Only for very low average degrees can it be observed that randomised SW graphs are, on average, 10% more efficient than the corresponding ER graphs. In SW graphs, $p_r$ and $deg_{avg}$ are both limiting factors for the convergence rate. Increasing $p_r$ rather than $deg_{avg}$ has the most effect on performance (Fig. 8d), meaning that rewiring existing edges is generally a more effective strategy than creating new edges.

#### BA graphs

Similar experiments on BA graphs show that $C_{r}$ depends on $deg_{avg}$ according to the same asymptotic model seen in ER graphs (Fig. 8e)

$$C_{r} = M_{BA} - b_{BA} \; e^{(- \; k_{BA} \;\; deg_{avg})},$$

but with a lower maximum rate ($M_{ER}$ = 0.490 vs. $M_{BA}$ = 0.450). The model describes data with high accuracy (r-squared = 0.985) and fitting parameters $M_{BA}$ = 0.450 [0.449, 0.451], $k_{BA}$ = 0.149 [0.143, 0.155], $b_{BA}$ = 0.534 [0.510, 0.558]. BA and ER graphs have comparable performance for $deg_{avg}$ below 15. However, ER graphs are, on average, 10% faster than BA graphs for $deg_{avg}$ above 15. Moreover, unlike ER graphs, BA graphs can never reach the maximum speed observed in complete graphs. ER and BA graphs are both characterised by highly randomised edges, low clustering coefficient and low shortest path. The main difference between the two families lies in the degree distribution: ER graphs have a narrow degree variance that depends on $deg_{avg}$, while BA graphs can have an arbitrarily large degree variance. The results suggest that a lower variance yields better performance for equal average degrees.

#### SF graphs

In SF graphs with adjusted clustering, the convergence rate is close to that of the corresponding BA graph for $p_c$ up to 0.8, while it greatly declines for $p_c$ closer to 1 (Fig. 8f). In these graphs, the average degree calculated solely considering preferentially attached (PA) edges named $deg_{pa}$ is most predictive of the convergence rate, while the $deg_{avg}$ alone has little explanatory power. In fact, in SF graphs, preferentially attached edges are responsible for propagating the information across the graphs, while triangle-forming edges mainly transmit redundant information. Similarly, rewired edges are most important for effective communication and convergence in SW graphs. The exponential model is similar to that for BA graphs (Fig. 8h)

$$C_{r} = M_{SF} - b_{SF} \; e^{(- \; k_{SF} \;\; deg_{pa})},$$

with estimated parameters $M_{SF}$ = 0.435 [0.434, 0.436], $k_{SF}$ = 0.478 [0.465, 0.490], $b_{SF}$ = 1.073 [1.039, 1.108], and record good explanatory value (r-squared = 0.876). It has a lower maximum rate ($M_{BA}$ = 0.450 vs. $M_{SF}$ = 0.435) but faster rate of increase ($k_{BA}$ = 0.149 vs. $k_{SF}$ = 0.478) compared to the BA model. For small values of $deg_{pa}$, the SF graphs perform better than the BA counterparts, meaning that, for very sparse scale-free graphs, adding a triangle-forming link increases $C_r$ (Fig. 8g).

#### GR graphs

Simulations on GR graphs found that $C_r$ grows linearly with the radius squared (Fig. 8i). Unlike the previous cases, $deg_{avg}$ alone has little predictive power on the graph behaviour. Considering that the radius is generally set as a multiple of $\sqrt{\frac{log(n)}{n}}$ to guarantee convergence and reduce interference, the time and number of messages required to converge to the global average with error *e* are then respectively bound by $\Theta (\frac{n \; log(e^{-1})}{log(n)} )$ and $\Theta (\frac{n^2 \; log(e^{-1})}{log(n)} )$, as previously shown^2^.

#### Position of starting values

The ER and SW graphs generated in this investigation are homogeneous by design, so the position of the values assigned to each node is unlikely to affect the overall convergence rate of the graph. However, similar considerations cannot be made for SF and GR graphs. Simulations are performed with and without shuffling the initial vector to distinguish the variance of $C_r$ caused by random interactions from that associated with the initial assignment. For ER, SW and SF graphs, the standard deviation of the rates in the two groups is not significantly different for all combinations of parameters, suggesting that the observed variance is primarily due to the random sequence of interactions. In GR graphs, however, the initial assignment significantly affects rates when the graph is very sparse. For instance, in a GR graph of size 500, the standard deviation in the shuffled group is 20 times higher at $deg_{avg}$ equal to 7 (the minimum degree that guarantees convergence), 10 times higher at a $deg_{avg}$ of 17, and similar to that of the unshuffled group for $deg_{avg}$ of 27 and above.

### Predictive power of individual graph metrics on algorithm performance

**Figure 9:**
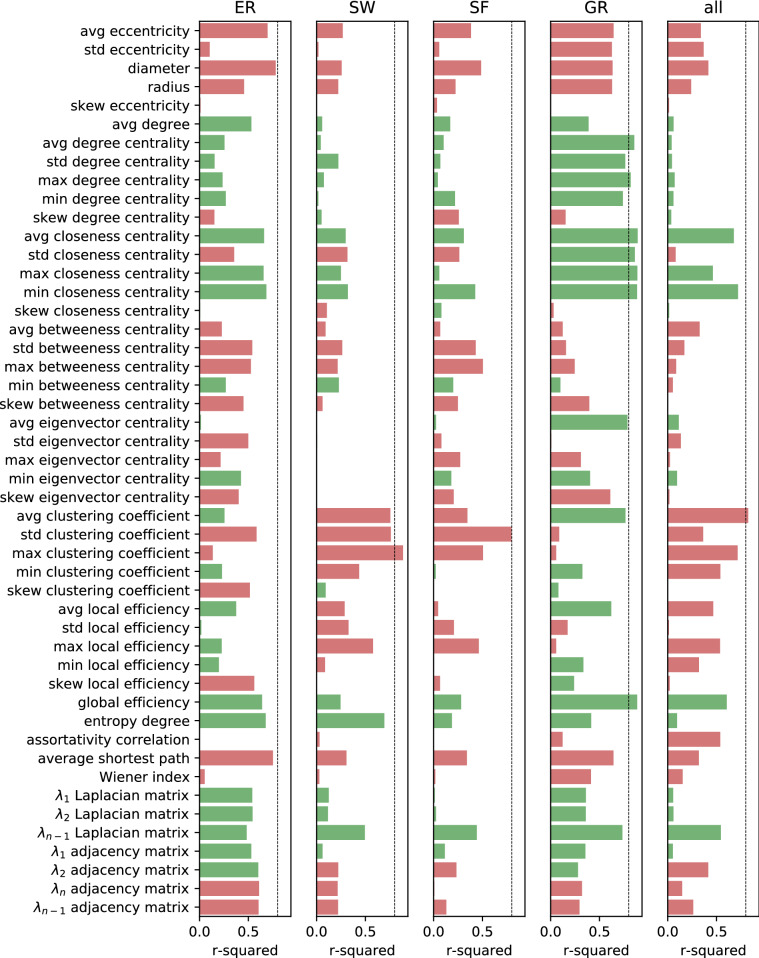
Linear regression r-squared values of graph metrics versus convergence rate for each graph family and for all graphs, coloured in red if the regression coefficient is negative and green if positive. The dotted line indicates an r-squared value of 0.8.

**Figure 10:**
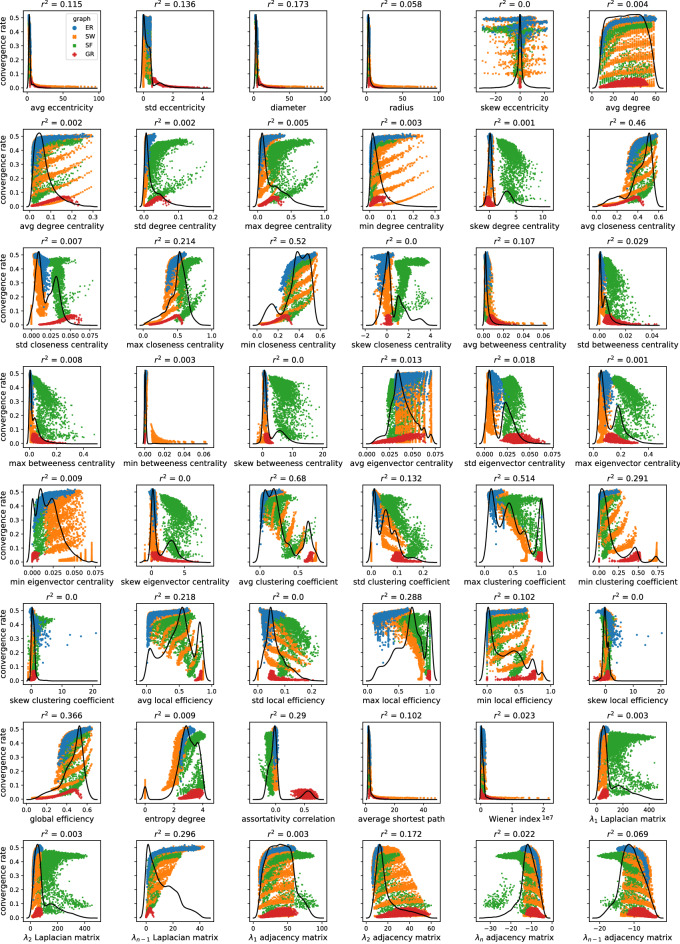
Scatter plots of graph metrics versus convergence rate in ER (blue), SW (orange), SF (green) and GR (red) graphs. The solid black line represents the distribution of the metric across all graph families.

A comprehensive set of global, local and spectral metrics quantifying relevant topological features is calculated for a sample of over 12,000 sparse networks. Linear regression analysis is performed between each metric and the convergence rate of the random gossip algorithm to estimate their predictive value in each family and all graphs (Fig. 9). A visual representation of the distribution of each metric in the four families and its explanatory power over the convergence rate is provided in Fig. 10. GR graphs have several parameters holding high explanatory power, while ER, SF and SW graphs only have a handful. Degree centrality, closeness centrality, and eccentricity metrics are predictive in GR graphs because they depend on the radius, which determines the convergence rate. Global metrics of distance (diameter, average shortest path, average eccentricity, closeness centrality) are most predictive for ER graphs, with the diameter holding the highest explanatory power. In SW graphs, maximum clustering negatively correlates with $C_r$; that is, nodes making many connections with their neighbours (and few with other nodes) are limiting factors for the convergence rate. Conversely, the entropy degree positively correlates with $C_r$, confirming that convergence speeds up as the graph deviates from the regular lattice structure. Spectral and eccentricity metrics, as well as degree, eigenvector and betweenness centrality, hold very little predictive value. In SF graphs, the clustering coefficient is the most predictive metric since higher clustering corresponds to fewer preferentially attached edges and slower convergence. Most metrics either positively or negatively affect $C_r$ in all graph families. Eccentricity metrics, average shortest path and Wiener index negatively correlate with $C_r$ in all experiments, suggesting that the distance within the graph is always a rate-limiting factor regardless of the graph topology. All degree metrics except the skewness positively correlate with $C_r$, confirming that an increase in the graph density yields a faster convergence. The average clustering coefficient has a positive correlation coefficient with $C_r$ in ER and GR graphs, where it is a measure of density, but negative in SF and SW graphs, where high clustering corresponds to insufficient edge randomisation. Entropy degree and global efficiency, which measure the information contained within the network and the ability to propagate it, record positive correlation coefficients across all families. Closeness centrality is consistently associated with a faster convergence rate and is the only centrality metric holding explanatory value for all graphs. Generally, a single metric has little predictive power over all graph families as they display vastly different topological profiles, while a combination of various metrics would allow for better predictions.

### Predictive power of sets of metrics on algorithm performance

A multi-linear regression model of all metrics records an adjusted r-squared of 0.987 but suffers from possible multicollinearity and over-fitting due to the high number of variables and degree of correlation among them. Univariate linear regression and mutual information tests are used to estimate the effect of each regressor on the target and help identify a handful of variables that account for the most variability. The distributions shown in Fig. 10 also suggest that several features are unlikely to hold significant explanatory value. For instance, most skewness metrics have very narrow or bimodal distributions, and removing them does not affect the model performance (0.986). Spectral metrics considered individually never achieve an r-squared above 0.6 but together account for over 78% of the variability in the data. However, their removal does not substantially affect the model’s explanatory power (0.981), suggesting that the information is also encoded in other features. In very large graphs, it is preferable to adopt non-spectral metrics because eigenvector calculations become time-consuming or even unfeasible. Distance and eccentricity metrics are highly skewed because the SW graphs with low rewiring probability have much larger internal distances, resembling a regular lattice. They record low r-squared scores (0.446 and 0.447, respectively, 0.454 together) and removing them from the model does not affect its performance (0.982). All degree metrics score low individual r-squared values, with entropy degree being the lowest (0.052) and assortativity correlation the highest (0.365), so that they can be safely removed (0.978). Global efficiency and local efficiency metrics score respectively 0.455 and 0.565, but when considered together, account for over 85% of the variability in the data, proving that global and local versions of the same metrics do not offer identical contributions. These metrics do not hold unique information since removing them barely affects the overall score (0.975). Centrality metrics are also expected to contain redundant information, and only the most relevant should be selected. The most predictive centrality is closeness (0.671), followed by betweenness (0.576), eigenvector (0.301) and degree (0.222). For these metrics, the minimum values hold the most explanatory power (0.828), followed by average (0.692), maximum (0.680), standard deviation (0.493) and skewness (0.481), confirming that the least central nodes constitute limiting factors for convergence. Clustering metrics have the highest predictive power (0.757) and, combined with the sole closeness centrality, recover an r-squared of 0.949. Global and local metrics hold comparable predictive power (95% and 92%, respectively), but the latter have considerably lower computational complexity, especially for sparse graphs, and can be calculated in a distributed manner and in constant time.

### Local prediction of convergence rate

A linear regression model of local metrics averages (average degree, average local clustering and average local efficiency) retains 91.4% accuracy. These results suggest that if nodes could estimate the average of these local metrics, they could also make predictions of the graph convergence rate $C_r$. Let *R* be a measure of accuracy such that the error at time *t* has been reduced by *R* orders of magnitude, i.e.

$$R = log\left( \frac{\Vert {\textbf {err}}(0) \Vert }{\Vert {\textbf {err}}(t) \Vert }\right) $$

**Figure 11:**
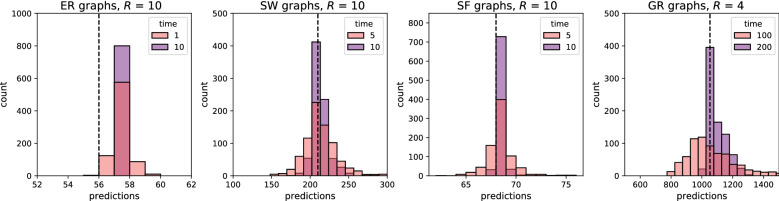
Distributions of expected times to achieve the desired accuracy *R*, predicted by each node using estimates of average local metrics. The dotted line indicates the actual time the condition was satisfied in simulations. The four graphs belong to different families, but all have $n = 800$ and $deg_{avg}=16$. The SW network has a $p_r = 0.125$, while the SF network has $p_c = 0.75$. The graphs include predictions made at two different time points to show how they become more accurate over time.

If nodes make one interaction per unit of time, i.e. $q = 1$, then the time taken to reach the desired level of accuracy *R* is equal to $R/ C_r$. These considerations motivate the design of a modified simulator where nodes propagate local metrics, estimate their average by distributed averaging and calculate the convergence rate. Nodes compute the considered local metrics by gathering the necessary information from immediate neighbours. When interacting with neighbours, nodes communicate their local metrics together with the measured quantity with little overhead and update their estimates by averaging them with their neighbours. Nodes then plug in the metrics in the regression model and obtain the time needed to achieve the desired level of accuracy, which could vary between nodes. Figure 11 shows the distribution of predictions made by nodes of the time necessary to reduce the error by $10^{10}$ in ER, SW and SF graphs (*R* = 10) and by $10^{4}$ in GR graphs (*R* = 4). Predictions are fairly accurate and improve with time, as they normally distribute around a value very close to the actual time, with variance decreasing over time. Nodes appear to make predictions of accuracy at time *t* well before that time elapses, e.g. a GR graph predicts at time $t = 200$ that the value will be sufficiently accurate at $t = 1100$ as shown in Fig. 11. The prediction quality necessarily depends on the convergence rate. Predicting convergence rates in geometric random graphs is particularly challenging because the propagation of local metrics is slow, and rates are largely affected by the initial position of the values, which the current model does not address.

### Comparison of gossip algorithms

**Figure 12:**
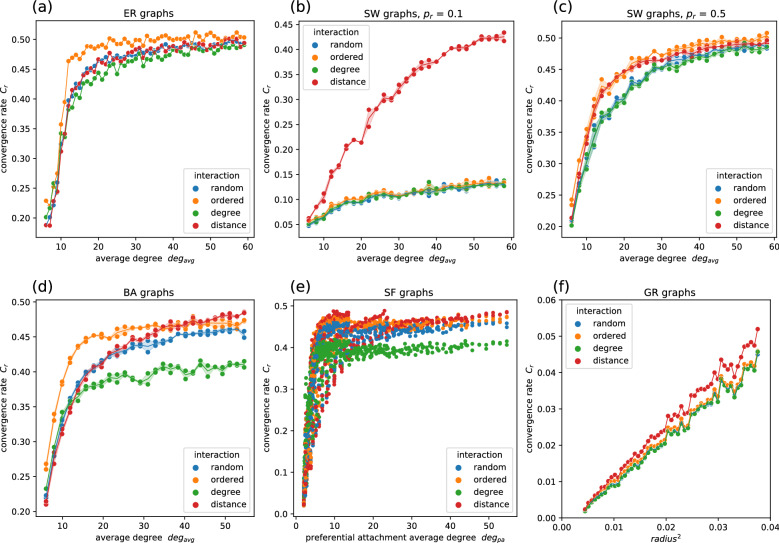
Performance of four gossip algorithms in (**a**) ER graphs, (**b**) SW graphs with low $p_r$, (**c**) SW graphs with high $p_r$, (**d**) BA graphs, (**e**) SF graphs with adjusted clustering, and (**f**) GR graphs. Each convergence value is the average convergence rate obtained over 100 simulation runs.

The convergence rate of a random gossip algorithm is compared with that of gossip schemes applying different neighbour selection criteria: ordered selection follows a fixed order to guarantee an equal number of interactions with each neighbour; degree selection preferentially chooses more connected nodes because more likely to hold new information; distance selection prefers nodes with fewer common neighbours to reduce the propagation of redundant information. In ER graphs, the ordered selection is the most effective, especially for values of $deg_{avg}$ between 10 and 30 (Fig. 12a). It achieves a rate of 0.45, close to the maximum rate of 0.50, at a $deg_{avg}$ of 12, while random, degree and distance selections require an average degree of 19, 23, and 20, respectively. ER graphs have homogeneous nodes and narrow degree distribution, so selecting a node over another based on slight differences in degree or distance is not an effective strategy. Similarly, the ordered selection is most effective in BA graphs, where it achieves a $C_r$ of 0.45 at a $deg_{avg}$ of 17, whereas distance and random selection reach a comparable $C_r$ at double the average degree (Fig. 12d). Degree selection is the least-performing algorithm in BA graphs, as it converges to a maximum rate of 0.42, well below that of all other algorithms ($\approx$ 0.48). In BA graphs, the high skewness of the degree distribution results in a few hubs being disproportionally selected for interaction and other less-connected nodes never being chosen, so fast convergence cannot be achieved. In SF graphs, degree selection remains the least effective algorithm, while the others yield comparable convergence rates (Fig. 12e). The performance of distance selection in SF graphs also depends on the clustering probability: it is faster than random selection at high clustering and slower otherwise. In GR graphs, also characterised by high clustering, distance selection yields higher $C_r$ than the other algorithms, although of only about 5% (Fig. 12f). In SW graphs, $C_r$ of distance selection is many folds higher than that of other schemes in less randomised graphs ($p_r < 0.3$), as shown in Fig. 12b. The other algorithms become faster as rewiring increases and approximate the behaviour of ER graphs when almost all edges have been randomised ($p_r > 0.5$), as seen in Fig. 12c. It appears that degree selection does not offer any advantage regardless of the skewness of the degree distribution. Distance selection is most effective when clustering is high, while random selection is preferable when the graph is sufficiently randomised. Ordered selection is fastest in randomised structures with low average degrees (below 30) as it prevents close same-node interactions.

## Discussion

The study analyses the convergence rate of random gossip algorithms in the four most popular models of real-life networked systems. The numerical experiments identify the average degree as the most predictive feature for convergence rate in most graph families. More connected graphs are expected to communicate more efficiently and perform faster distributed computations. However, the relationship is non-linear and best approximated by an asymptotic exponential trend. According to this model, increasing the average degree boosts performance in very sparse graphs but has almost no effect in denser graphs. Establishing new connections requires resources in every scenario or application; otherwise, each node would simply connect with all other nodes in the network. In wireless sensor networks, for instance, more links can be created by increasing the communication range, although these transmissions are more energy intensive. Hence, in gossiping algorithms, there is a trade-off between the desired performance and the required resources. The regression results can help decide whether the predicted performance increase justifies the added cost of creating new connections. Similarly, in conditions of unstable communication, they prescribe how many links should remain active to guarantee performance. In ER graphs, often adopted to model networks with unreliable communication, 99% of the maximum convergence is preserved if each node retains about 28 neighbours but decays exponentially below that, so effort should be put toward maintaining the average degree above this level.

The simulations also highlight the opposite effects of clustering and rewiring on performance. In SW graphs, increasing the graph density has minimal impact on performance compared to edge rewiring. These graphs are often adopted to model social networks and opinion dynamics. The results suggest that resources should be put toward promoting the formation of random connections to achieve faster consensus in the population. In SF graphs, clustering determines the overall stability and reliability of the network. Increasing clustering has a limited effect on performance up to a rewiring probability of 0.8, but it significantly reduces it above that. In these graphs, edges generated by preferential attachment are responsible for most information transmission, while triangle-forming edges propagate redundant information. Hence, there is also a trade-off between performance and stability.

The analysis of graph metrics in sparse graphs unveils the effect of various topological features on convergence. The regression analysis identifies closeness centrality and clustering coefficient as the two most predictive features, accounting for 95% of the variability in the data. In performing graphs, the distance between each node and any other node is relatively low, i.e. each node can communicate with any other node in a few hops. Moreover, each node shares few connections with any of its neighbours, limiting the propagation of redundant information. Local metrics hold a high predictive power (92%), require fewer computational resources, and can be fully parallelised. A model propagating local metrics exemplified how nodes can exploit distributed averaging to estimate both the population average and its accuracy so each node can use, process, or communicate its estimate only when confident of its accuracy. Finally, a comparison of the performance of four gossip algorithms showed that distance selection and ordered selection can boost convergence in highly clustered and very sparse structures, respectively.

The multiple findings and insights in this study demonstrate the value of graph measures in investigating and predicting performance on networks. Such topological approaches are well-established and widely adopted in the study of network robustness and security but are still fairly unexplored in distributed computation. This work aims to bring attention to this line of research and inspire further investigation in this direction. Future work entails repeating the analysis on larger graphs (*n*> 10,000) and extending it to other topologies often found in real networks, first and foremost community-based structures, which are sparsely connected on a large scale but highly connected on a small scale. Time-varying topologies should be considered to address intermittent information transmission, switching communication topology, and time-varying communication delays that characterise distributed networks^16^. Heterogeneous models, with nodes adopting different interaction rates, time delays or neighbours selection criteria, can also be investigated within the same framework^58^. Cooperative and non-cooperative game theoretic formulation of the node behaviour can also be explored, as previously suggested^59^. Deterministic gossip in which nodes interact with neighbours according to a given sequence, such as the ordered selection considered in this investigation, can boost convergence in given topologies and should be further analysed^60^. Finally, more accurate predictive models of convergence rate can be obtained by including maxima, minima and linear statistics, which nodes can propagate according to the same distributed averaging mechanism and use to determine convergence.

## Conclusions

This study adopts an experimental approach to characterise the topological determinants of the convergence rate in gossip algorithms. The numerical experiments uncover the asymptotic relationship between graph density and convergence rate and highlight the effect of clustering and rewiring on performance in four representative graph families. These results can inform topological interventions, as well as the design and maintenance of resource-efficient networks performing distributed computation. In contrast, the comparison of gossip schemes reveals how certain network configurations can benefit from an alternative node selection criterion. The study also investigates the predictive capabilities of graph metrics, which quantify global and local topological properties of networks. Closeness centrality and clustering coefficient are identified as the most predictive metrics across all graph families, suggesting that, in efficient graphs, nodes are relatively close to all other nodes but have few shared connections with their neighbours. Regression analysis reveals that selected metrics can reliably predict the convergence rate on a given graph, demonstrating that topology determines performance and confirming the validity of the chosen approach. The high predictive power of local metrics and the possibility of computing them locally at a low computational cost motivates the design of systems estimating performance based on the network topology and in a fully distributed fashion. Numerical experiments where nodes propagate local metrics, estimate their average by distributed averaging, and accurately predict the convergence rate confirm the validity of this approach.

## Data Availability

The datasets generated and analysed during the current study are available in the GitHub repository, https://github.com/ChristelSirocchi/topology_convergence.
